# Effects of dexmedetomidine on stress hormones in patients undergoing cardiac valve replacement: a randomized controlled trial

**DOI:** 10.1186/s12871-020-00993-0

**Published:** 2020-06-06

**Authors:** Hanhua Wu, Jinqing Tang, Jiamei Pan, Ming Han, Huijun Cai, Hong Zhang

**Affiliations:** 1 Department of Anesthesiology, Affiliated Hospital of Zunyi Medical University, No. 149 Dalian Road, Huichuan District, Zunyi, 563003 Guizhou Province China; 2 Department of Anesthesiology, Third Affiliated Hospital of Zunyi Medical University, Zunyi, 563003 Guizhou Province China

**Keywords:** Dexmedetomidine, Cardiopulmonary bypass, Hormones

## Abstract

**Background:**

Stress response always occurs in cardiac valve replacement patients undergoing cardiopulmonary bypass (CPB).

**Methods:**

60 patients undergoing cardiac valve replacement were recruited and randomized into control and Dex groups. Dex group received 1.0 μg·kg-1 of Dex for 10 min intravenously before anesthesia, followed by 0.5 μg·kg-1·h-1 of Dex, steadily administered throughout the procedure. And controlled group received the identical velocity of saline as Dex group. Plasma level of cortisol (Cor), epinephrine (E), norepinephrine (NE), and serotonin (5-HT) were evaluated at four timepoints: Before administration (T0), sawn sternum (T1), end of extracorporeal circulation (T2), and 24 h post operation (T3). General data of operation and recovery such as heart rate (HR), mean arterial pressure (MAP), intraoperative bispectral index (BIS), and hospitalization time in the intensive care unit (ICU) were also compared.

**Results:**

Increase of Cor, E, NE, and 5-HT for the Dex group was significant lesser than that in the control group (*P* < 0.05), and ICU hospitalization time and ventilator support time was significantly shorter in the Dex group. The proportion of patients discharged from the hospital with better prognosis was significantly higher than that in the control group, while there were no significant differences in hospitalization costs and vasoactive drugs use between the two groups.

**Conclusions:**

Dex reduces plasma Cor, E and NE elevations in patients after CPB, alleviates the stress reaction of the body, shortens the hospitalization time and ventilator support time in ICU, and plays a positive role in the rehabilitation of patients undergoing cardiac valve replacement.

**Trial registration:**

China Clinical Trial Registry (No. ChiCTR-IPR-17010954) March 22rd, 2017.

## Background

Cardiopulmonary bypass (CPB) immensely facilitated open heart surgical procedures such as cardiac valve replacement, benefitting patients for decades [1]. However, procedural exposure to a non-physiological condition, elicited a strong neuroendocrine stress response in patients due to several reasons such as cardiac arrest, blood dilution, and ischemia-reperfusion injury [2]. Generally, the serum hormones have a physiological circadian rhythm, with a peak in the morning between 8 and 9 a.m. Surgical stress response may be a major causative factor leading to physiological and biochemical disturbance of the homeostatic axes, primarily affecting the hypothalamic-pituitary-adrenal cortex (HPA) and locus coeruleus-sympathetic-adrenal medullary system [3]. Previously, changes in secretion of HPA related hormones such as corticotropin releasing hormone (CRH), adrenocorticotropic hormone (ACTH), glucocorticoid (GC) and Cortisol (Cor) were observed [4]. Similarly changes in the locus system related hormones such as noradrenaline (NE), epinephrine (E) and dopamine (DA) were observed [5, 6].

Increased secretion of surgical stress response associated hormones could cause tachycardia and elevate myocardial oxygen demand, peripheral vascular resistance, and oxygen consumption. Moreover, α-receptor activated sympathetic nerve stimulation may also lead to contraction of the coronary artery inducing myocardial ischemia [7]. Clinically, administration of anesthetic drugs to alleviate stress, could be effective in decreasing postoperative complications and mortality [8]. Dexmedetomidine hydrochloride (Dex) is a highly specific and selective α2 adrenergic receptor agonist (α2-AR), having the following physiological effects: sedation, analgesia, inhibition of sympathetic nerve, alleviation of inflammatory response, and reduction of stress response [9]. Moreover, Dex was effective for blunting the elevation in systolic blood pressure and stabilizing heart rate perioperatively. And it had little adverse effect [10].

We aimed to investigate if Dex can alleviate intraoperative stress response and accelerate patient recovery by observing its effects on Cor, E, NE, and 5-HT concentration in plasma of patients undergoing cardiac valve replacement.

## Methods

### Study design

This was a randomized controlled clinical trial to evaluate effects of Dex administration in order to control intraoperative stress response, by observing changes in hormone levels at different timepoints. The trial adheres to CONSORT guidelines and was approved by the Ethics Committee of the First Affiliated Hospital of Zunyi Medical University and registered in the China Clinical Trial Registry (Registration No. ChiCTR-IPR-17010954).

### Participants

We selected patients who underwent cardiac valve replacement between 9:00 and 11:00 am, from March 2016 to April 2017, in the First Affiliated Hospital of Zunyi Medical University.

Inclusion criteria: (1) Age, 40–65 years; (2) American Society of Anesthesiologists (ASA) physical status classification of II or III; (3) expected operative time less than 5 h; (4) altitude of permanent residence less than 2500 m; (5) absence of symptoms of rheumatic fever (RF < 20).

Exclusion criteria: (1) Patients who discontinued anticoagulants for less than 3 days before operation; (2) Second cardiac surgery, (3) Hypertension (systolic blood pressure ≥ 180 mmHg or diastolic blood pressure ≥ 110 mmHg), anemia (hemoglobin less than 7.0 g/L), low blood volume or low protein (< 30 g/L); (4) Sinus node dysfunction with impaired conduction of impulses; (5) Severe liver dysfunction (AST > 120 U/L or ALT> 129 U/L), kidney dysfunction (SCR ≥ 150 μmol/L) and coagulation disorders (INR < 1.8 or INR > 2.0 for aortic valve replacement patients, INR < 2.0 or INR > 2.5 for mitral valve replacement patients, INR < 2.5 or INR > 3.0 for tricuspid valve replacement patients); (6) Severe pulmonary infections and diseases (imaging results of pulmonary inflammation or respiratory symptoms such as coughing); (7) Heparin resistance and allergy in patients.

Termination criteria: (1) Uncontrollable procedural emergencies; (2) If the patient’s condition was serious and they could not be successfully rescued during or 24 h after surgery; (3) Re-cardiopulmonary bypass or surgery; (4) Difficulty in cardiac resuscitation (defibrillation > 4 beats) and cardiopulmonary bypass stopping; (5) Surgical procedures that could develop sever stress response such as intraaortic balloon pump, extracorporeal membrane oxygenation (ECMO), and deep hypothermic circulatory arrest.

**Fig. 1:**
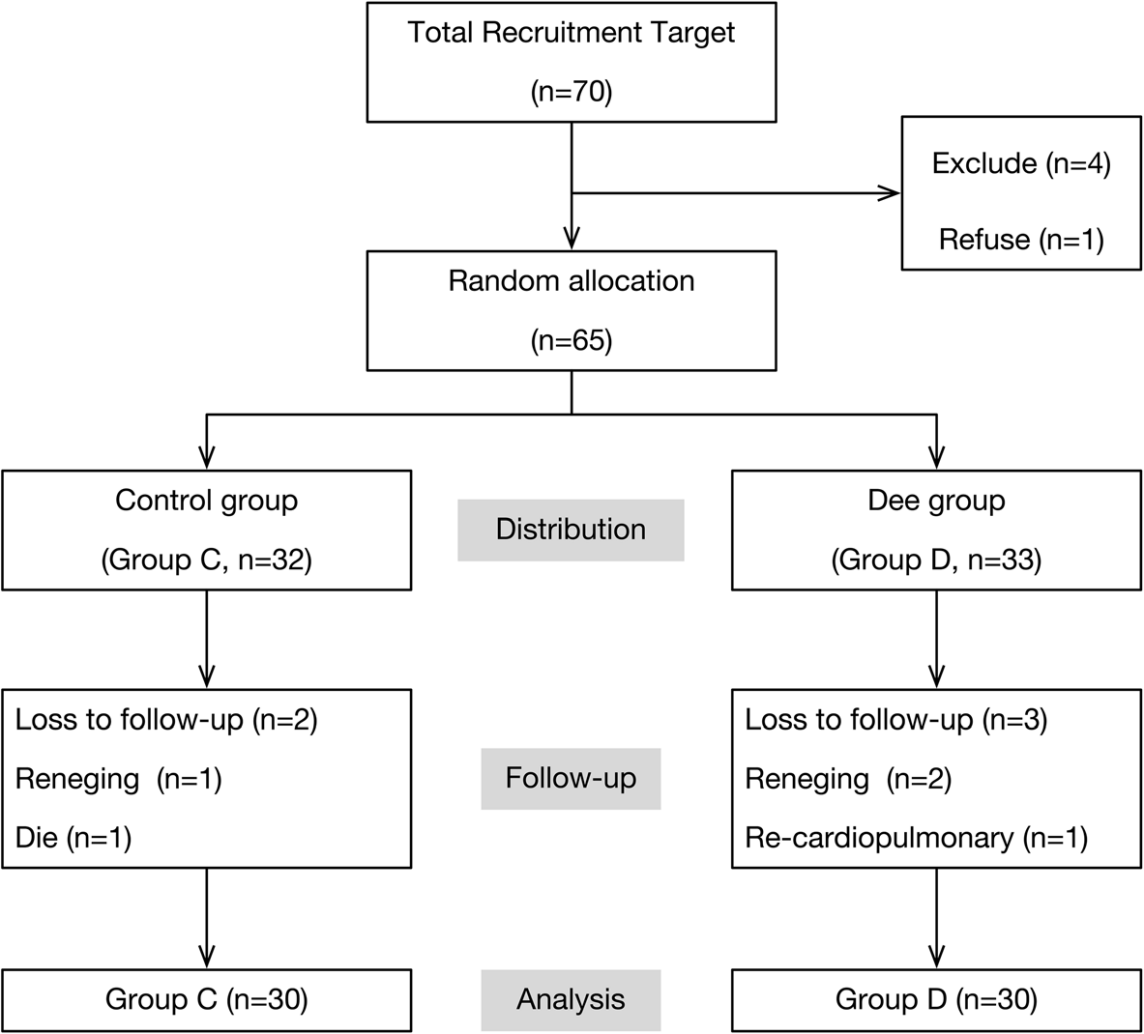
Study flow chart

The flow-diagram of participant selection is summarized in Fig. 1. Data of 60 patients were analyzed in this study.

### Procedures and assessments

Participants received detailed information about this study and provided informed consent before baseline assessment, following which, study assistants opened a prepared sealed envelope containing group allocation information. Participants were randomly allocated to either a Dex group (0.2 mg Dex extracted to 50 mL normal saline) or control group (50 mL normal saline). In the Dex group (Group D), 1.0 μg·kg^− 1^ Dex was infused intravenously for 10 min before inducing anesthesia. Subsequently, 0.5 μg·kg^− 1^·h^− 1^ Dex was administered for maintenance until the end of operation. Participants in the control group (Group C) were administered saline at an infusion velocity identical to that of the Dex group, in the induction and maintenance phase of the procedure.

Demographic features such as gender, age, weight, ASA level and Euroscore were collected before the surgery. During the operation, the CPB transit time, ascending aorta occlusion time, operative time and condition of cardiac resuscitation (automatic cardiac resuscitation or cardiac resuscitation after defibrillation) were recorded. Moreover, four timepoints were chosen for testing the heart rate (HR), mean arterial pressure (MAP), bispectral index (BIS) and concentration of hormones in the blood as follows: before administration (T0), at sternum sawing (T1), at end of extracorporeal circulation (T2) and 24 h after operation (T3). Five milliliters of blood sample of each patients were tested for the level of Cor, E, NE and 5-HT. After the surgery, the hospitalization time in ICU, ventilator support time, condition of improvement and hospitalization expense were recorded and compared between two groups. The different vasoactive drugs use (during the operation and after the operation) was also recorded and compared between two groups of patients. Patients with improvement were defined as improvement of cardiac function, no abnormity of valve replacement, normal coagulation function and general condition. The primary outcome of this study were the plasma Cor, E, NE and 5-HT level at T3, while the concentration at other timepoints were considered as secondary outcomes. In addition, the secondary outcomes included HR, MAP and BIS at all follow-ups, the ICU hospitalization time, ventilator support time, proportion of patients with improvement and discharged from hospital, hospitalization costs and the intraoperative and postoperative vasoactive drug dosage.

### Laboratory analysis

Cor in plasma was measured using an enzyme-linked immunosorbent assay (ELISA) kit (Shanghai Jianglai Biotechnology Co., Ltd. Shanghai). We evaluated E, NE, and 5-HT in plasma by High Performance Liquid Chromatography (HPLC) Using Agilent 1200 high performance liquid chromatography (Agilent Company, USA). The purity of E, NE, and 5-HT standard products was higher than 99%, purchased from Sigma Chemical Reagent Company. E was recovered by 100–117%. NE and 5-HT were successfully recovered by 100–110%, respectively, and relative standard deviation of less than 5%. The normal range for Cor was 20-250 ng/mL. Normal ranges were 0.30–3.00 pg/μL for E, 0.36–1.98 pg/μL for NE and 0.97–2.09 pg/μL for 5-HT.

### Anesthesia induction and cardiopulmonary bypass management

Anesthesia induction was achieved with midazolam (0.06–0.1 mg·kg^− 1^), sufentanil (1–3 μg·kg^− 1^), etomidate fat emulsion (0.15–0.3 mg·kg^− 1^), and rocuronium (1 mg·kg^− 1^). Continuous propofol (4–12 mg·kg^− 1^·h^− 1^) and sufentanil (1–2 μg·kg^− 1^·h^− 1^) infusions were administered intraoperatively to maintain anesthesia and muscle relaxation, and inhalational anesthetics such as sevoflurane were also used in certain situation. To ensure the safety of our patients, we kept the hemodynamics and BIS in a safe range. In order to maintain effective visceral perfusion, the MAP was controlled between 50 and 80 mmHg, and the HR was maintained between 60 and 120 bpm. The BIS was maintained over 45 during anesthesia induction. Then the BIS was maintained between 40 and 60 during the surgery. Moreover, we also administered routine adjuvant drugs such as omeprazole, ulinastatin, and tranexamic acid.

Here, we used the cardiopulmonary bypass machine (C5, Sorin; Munich, Germany), hollow fiber super filter (0.8m^2^, MicroPort, Dongguan, China), CPB pipeline (adult, TPRI; Tianjin, China), BIS monitoring module monitor (BeneViewT5, Mandary; Shenzhen, China) and membrane oxygenator (541,Medtronic, Memphis, United States). Crystal and colloid liquid ratio of CPB pre-filling fluid was approximately 1:1. Hematocrit (Hct) was maintained between 21 and 25%, and suspended red blood cells were administered as required. Pre-filling fluid volume was approximately 1700–2000 mL, and cardiopulmonary bypass was conducted during low body temperature and medium-high flow. After routine disinfection and laying the towel, skin incision, thoracotomy, 3 mg·kg^− 1^ heparinization, and pericardiectomy were carried out through a median incision. CPB was established by connecting the superior and inferior vena cava and ascending aorta with the corresponding pipelines of the pre-filled artificial cardiopulmonary machine. We used a perfusion needle to fix the aortic root, followed by connecting a myocardial protective fluid perfusion device. We commenced CPB after activated prothrombin time (ACT) of > 480 s. During CPB, we maintained the nasopharyngeal temperature at 32–34 °C, Hct at 25–30%, perfusion flow between 50 and 100 mL·min^− 1^·kg^− 1^, and MAP at 50–80 mmHg. Furthermore, the ion-acid-base balance was maintained according to blood gas analysis. Both biological and artificial valves were used in this study.

### Statistical analysis

For the sample size calculation, we considered a difference in E level of 1.0 pg/μL (δ) between the group treated with Dex and saline as clinically relevant and we specified such an effect to be detected with 90% power (0.9) and a significance level alpha of 0.05. For the population variance, our pilot study showed that in similar patients, the SD of E level was 1.3 pg/μL(σ). Then we used the formula *N* = 2 × [(α + β) σ / δ]^2^ to calculate the sample size, and the sample size was at least *N* = 28 patients in each group. The experimental data were analyzed by Statistical Package for the Social Sciences ver. 17.0 software (SPSS Inc., Chicago, IL). Measurement data were expressed by mean ± SD. Count variables were compared by the Chi squared test. Two independent sample t-test was used for inter-group comparison, and repeated measurement data variance analysis was used for intra-group comparison. We considered *P* < 0.05 as statistically significant.

## Results

Initially 70 patients were recruited for the study, of which 5 were excluded (4 did not meet the criteria and 1 refused to participate). After randomized allocation, there were 32 cases in the control group and 33 cases in the Dex group. During the follow-up, 2 patients refused the test and quit halfway, 1 patient was re-operated and 1 patient expired. Finally, a total of 60 patients were included in the analysis, including 30 in Group C and 30 in Group D (Fig. 1).

### Comparison of clinical data between the two groups

There were no significant differences in sex, age, weight, ASA category, Euroscore, CPB transit time, ascending aorta occlusion time, operative time, and cardiac resuscitation between the two groups (*P* > 0.05, Table 1).

**Table 1:** Comparison of clinical data between the two groups

Clinical data	Group C (*n* = 30)	Group D (*n* = 30)	t(×^2^)	*P*
Gender [M/F, n (%)]	19 (63)/11 (37)	16 (55)/14 (47)	0.617	0.432
Age (years)	51 ± 6	49 ± 4	1.420	0.161
Weight (kg)	55 ± 9	54 ± 8	0.015	0.988
ASA [II/III, n (%)]	5 (17)/25 (83)	9 (30)/21 (70)	1.491	0.222
Euroscore	2.03 ± 1.63	1.80 ± 1.58	0.562	0.576
CPB transit time (min)	115 ± 34	110.74 ± 37	0.502	0.618
Ascending aorta occlusion time (min)	73 ± 25	74 ± 36	0.150	0.881
Operative time (min)	247 ± 39	244 ± 49	0.261	0.795
Automatic cardiac resuscitation [YES/NO, n (%)]	2 (7)/28 (93)	3 (10)/27 (90)	0.218	0.640

### Comparison of HR, MAP, and BIS in the two groups

There were no differences in BIS values at identical time points between the two groups (*P* > 0.05). MAP at T2 was lower than that at T0 in both the groups (*P <* 0.05), HR at T1 in the Dex group was the lowest, but within the normal clinical range. HR and MAP of both groups had no significant differences among other timepoints. At T1, HR in Group D was significantly lower than that in Group C (*P* < 0.05), with no significant change in MAP. Additionally, there were no significant differences in HR and MA*P* values between the two groups at other timepoints (*P* > 0.05, Table 2).

**Table 2:** Comparison of HR, MAP and BIS in the two groups ($\overline{X}$ ±SD, *n* = 30)

Index	Group	T0	T1	T2	T3
HR (beats/min)	Group C	95 ± 23	98 ± 27	105 ± 20	103 ± 16
	Group D	94 ± 22	78 ± 14^a*^	108 ± 18^ab^	106 ± 16^ab^
MAP (mmHg)	Group C	87 ± 13	83 ± 12	70 ± 10^a^	78 ± 11^a^
	Group D	91 ± 10	80 ± 14^a^	74 ± 7^a^	82 ± 9^ac^
BIS	Group C	90.4 ± 1.7	37.9 ± 1.8	36.4 ± 1.7	–
	Group D	90.7 ± 1.8	38.3 ± 1.8	36.2 ± 1.7	–

*T0* baseline; *T1* at sternum sawing; *T2* at shutdown; *T3* 24 h after operation

* *P* < 0.05 as compared with group C

^*a*^*P* < 0.05 as compared with T0

^*b*^*P* < 0.05 as compared with T1

^*c*^*P* < 0.05 as compared with T2

### Comparison of plasma Cor, E, NE, and 5-HT levels in the two groups

In order to eliminate the influence of hemodilution after CPB on the results, Hct before CPB bypass was used as the standard reference. The formula was: correction value = measurement value * (Hct before bypass / Hct at the corresponding blood collection times).

Comparing the two groups at different times: Compared with T0, Cor, E and NE levels showed an increasing trend, while Cor and NE concentration in both groups at T1, T2, and T3 increased significantly (*P* < 0.05). The plasma Cor concentration at T2 and T3 in Group C was significantly higher than that at T1 (*P* < 0.05), while there were no significant differences for the same at T1, T2, and T3 in Group D. The plasma E concentration at T3 in Group C was significantly higher than that at T0 (*P* < 0.05), while there were no significant differences between the values at T1 and T2. There were no significant differences at T0, T1, T2, and T3 in group D. Plasma NE concentration at T0, T1, T2, and T3 in both groups, results showed a significantly increasing trend, while concentrations at each timepoint were significantly lower than the next timepoint (*P* < 0.05). The plasma 5-HT concentration at T2 and T3 was significantly higher than that at T0. When compared with T1, plasma 5-HT concentrations increased significantly at T2 and T3 (*P* < 0.05).

**Fig. 2:**
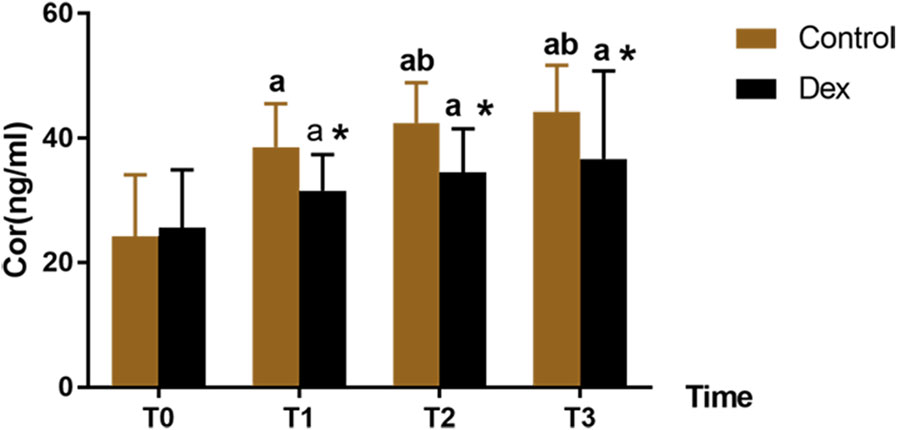
Comparison of Cor concentration at different timepoints between the two groups

**Fig. 3:**
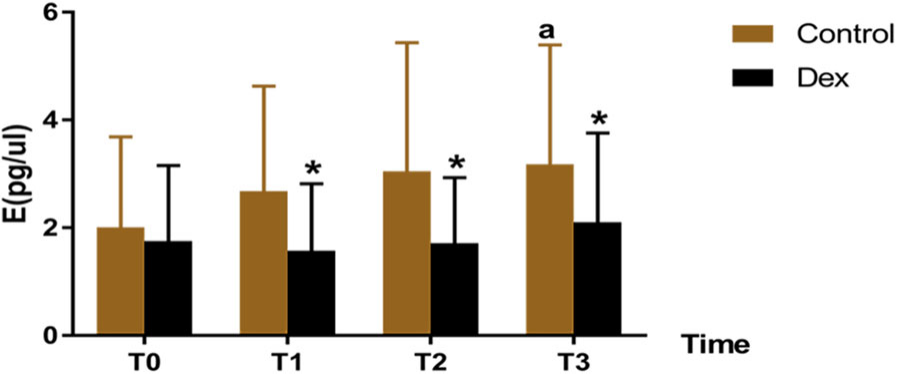
Comparison of plasma E concentration at different timepoints between the two groups

**Fig. 4:**
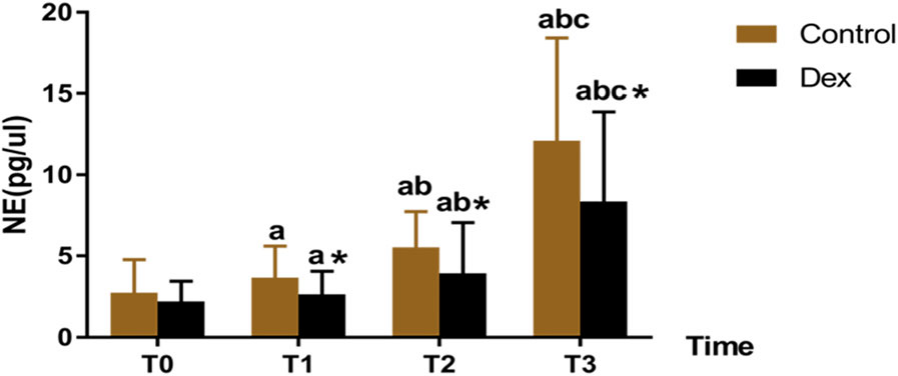
Comparison of plasma NE concentration at different timepoints between the two groups

**Fig. 5:**
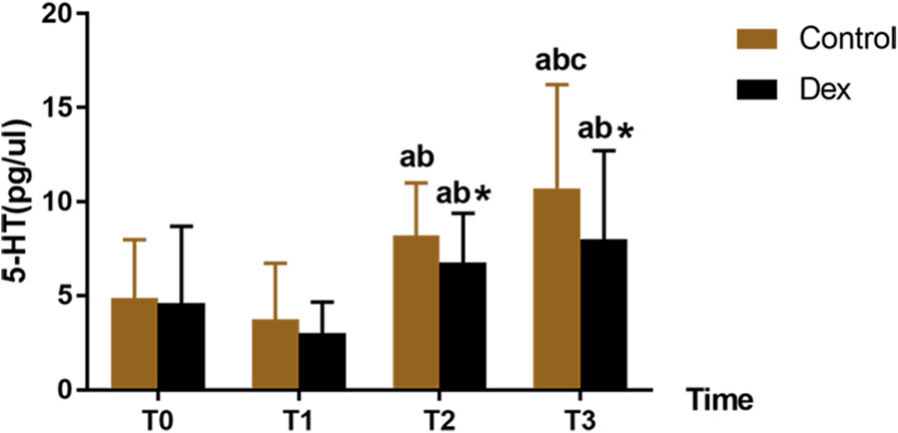
Comparison of plasma 5-HT concentration at different timepoints between the two groups. Different timepoints as follows: T0: baseline; T1: at sternum sawing; T2: at end of extracorporeal circulation; T3: 24 h after operation

At T0, there was no significant difference in plasma E, NE, 5-HT, and Cor for both groups (*P* > 0.05). At T1, T2, and T3, the plasma E, NE, and Cor levels in Group D were lower than those in Group C *(P* < 0.05). At T2 and T3, the plasma 5-HT levels in Group D were lower than those in Group C (*P* < 0.05, Figs. 2, 3, 4, and 5).

### Postoperative follow-up

ICU hospitalization time (*P* = 0.026) and ventilator support time (*P* = 0.042) were significantly shortened in Group D than in Group C (Table 3). Proportion of patients with improvement and discharged from hospital was significantly increased (*P* = 0.020). There were no significant differences in hospitalization costs and the intraoperative and postoperative vasoactive drug dosage between the two groups (*P* > 0.05, Table 3 and Table 4).

**Table 3:** Comparison of postoperative follow-up between the two groups ($\overline{X}$ ±SD, *n* = 30)

Postoperative follow-up	Group C	Group D	t(x^2^)	*P* value
Hospitalization time in ICU (h)	90.4 ± 72.0	55.4 ± 41.9	2.30	0.026
Ventilator support time (h)	44.5 ± 40.20	19.3 ± 16.3	2.11	0.042
Patients with improvement^△^ [YES/NO, n (%)]	25 (83)/5 (17)	30 (100)/0 (0)	5.46	0.020
Hospitalization expenses (Ten thousand yuan)	12.5 ± 5.1	11.4 ± 4.4	0.57	0.572

^△^ Patients with improvement were defined as improvement of cardiac function, no abnormity of valve replacement, normal coagulation function and general condition

**Table 4:** Comparison of dosage of vasoactive drugs used during and after the operation ($\overline{X}$ ±SD, mg)

Vasoactive drugs	Intraoperative dosage of vasoactive drugs (mg)			Postoperative dosage of vasoactive drugs (mg)		
	Group C	Group D	*P* value	Group C	Group D	*P* value
Adrenaline	0.55 ± 0.06	0.55 ± 0.07	0.798	0.97 ± 0.10	0.93 ± 0.17	0.272
Deoxyepinephrine	0.22 ± 0.18	0.22 ± 0.19	0.891	0.48 ± 0.34	0.47 ± 0.31	0.910
Dopamine	55.23 ± 7.10	55.00 ± 8.59	0.911	155.53 ± 18.42	155.44 ± 17.52	0.983
Glyceryl trinitrate	5.48 ± 0.74	5.46 ± 0.71	0.958	5.06 ± 0.68	4.99 ± 0.71	0.730

## Discussion

Our results showed plasma concentrations of Cor, E, NE, and 5-HT gradually increased with the prolongation of operation time. The plasma concentration of Cor, NE, and 5-HT at T2 was significantly higher than that at T0 and T1. Application of Dex preoperatively could significantly reduce plasma Cor, E, NE, and 5-HT concentrations than the control group, indicating that Dex could inhibit the stress response in patients undergoing valve replacement undergoing CPB. Moreover, 24-h follow-up showed that application of Dex could significantly improve ICU hospitalization time, ventilator support time, and rate of improvement and discharge, suggesting that it could be conducive to the recovery of patients who are undergoing CPB.

In our study, there was no difference between Dex and control group in terms of hemodynamics, and it might due to our study design. With regard to the MAP, Hashemian et al. found there was no difference between Dex and control group 0-2 h post CPB, and the difference of MAP was only found 1-2 h post operation [11]. Our study was partly in accordance with their results. However, the MAP of the control group was more than 80 mmHg 1-2 h post operation in their study, while we set our safe range of MAP between 50 and 80 mmHg. This unintentional control of HR and MAP might explain that we did not find any difference about HR and MAP between these two groups.

In healthy people, hormone secretion has a physiological circadian rhythm. For Cor, the peak of serum Cor concentration showed between 8 and 9 a.m. Then the concentration gradually decreased till 12–14 p.m. with a second peak during this period [12]. In our study, we chosen 9–11 a.m. to test the concentration of different hormones as for avoiding the impact of physiological circadian rhythm.

Recent study reported that the postoperative Cor levels did not show any difference between patients with and without CPB, which supported that CPB procedure did not specifically induce the stress response compared with other surgeries [13]. During the CPB procedure, an obvious increase of Cor and catecholamines level and presence of stress response have been reported in other studies [5, 13, 14], similar to our study. Cor is a stress response biomarker and is necessary in the defense reaction of the body [15]. Moreover, it has been reported that higher Cor levels are related to longer duration of stay, longer ventilator time, higher inotrope scores, and larger fluid requirement [16]. With regard to the effect of Dex on Cor released during operation, studies showed that it could inhibit Cor secretion [17]. However, there are studies which argue about the effects of short-term Dex usage in humans. Bekker et al. reported that Dex could have a negative impact on the plasma Cor levels [18]. However, Bulow et al. did not find any differences Cor levels while using Dex in the patients undergoing mini-CPB surgery [9]. In our study, we observed that patients in Dex group had a lower plasma Cor levels. Our study used etomidate for induction, which had been reported had negative effect on Cor level till 7 h postinduction [19]. However, both groups of patients received etomidate for induction, and we believed the difference on Cor level between Dex and control group could reflect the effect of Dex on plasma Cor level.

Considering plasma catecholamines levels, it has been reported that CPB is associated with the release of catecholamines. However, our results showed a slight increase in the E and significant change in the NE values after CPB, contrary to the result of a previous study, which indicated that the elevation of plasma E level was several times higher than the plasma NE level in patients after CPB [20]. This might be due to differences in the activation of the sympathetic nerve and adrenal medulla [21]. Plasma NE level is primarily affected by the activation of sympathetic system, which is particularly mediated by baroreceptor activation and physical issues. Additionally, hypothermia might significantly affect sympathetic nerve activation, resulting in increased plasma NE levels [20]. However, plasma E level is primarily associated with psychological stress and general and metabolic threat. Due to surgical intervention and the application of anesthetic drugs such as opioids, external nerve afferent impulse and neuroendocrine autonomic response might be blocked, resulting in an imbalances change of plasma E and NE levels [22].

Additionally, we observed that Dex had negatively affected both plasma E and NE levels than the control group, similar to a previous report, suggesting that the application of Dex could significantly lower the plasma Cor, E, NE, and blood glucose in pediatric cardiac surgery [23]. As the selective α2-AR agonist, Dex could specifically bind to presynaptic α2-AR and inhibit central sympathetic outflow trough a negative feedback mechanism. Due to the fact that these receptors are primarily located in the brain stem and locus coeruleus, Dex administration could significantly affect plasma NE levels [24]. Furthermore, a previous study also reported that Dex could suppress NE transporters by competitively inhibiting substrate transport [25]. With regard to the changes in the plasma E levels, it is our assumption that it might be due to the activation of both pre- and postsynaptic α ARs.

It has been reported that Dex could also inhibit the release of other neurotransmitters such as γ-aminobutyric acid, DA, and 5-HT in non-adrenergic neurons [26]. According to a previous animal study, 5-HT is involved in regulation of gene expression of hypothalamic hormones and the secretion of pituitary gland hormones. The study showed that 5-HT1A receptor activation promoted acetylcholine and NE release in brain and increased the blood concentration of CRH, ACTH, and Cor [27]. Dex administration could be significantly more beneficial in treating serotonin syndrome than the current treatment involving the use of benzodiazepines in rats [28]. There is also clinical evidence demonstrating that Dex could temporally stabilize the autonomic nervous system, improve agitation, and benefit successful extubation in three cases of severe serotonin syndrome [29]. Here, we also found Dex had a negative effect on the plasma 5-HT levels of patients who underwent CPB, which might indirectly contribute to decreased Cor and catecholamine levels. Moreover, it might have a direct effect on the central 5-HT system, resulting in improved recovery after the procedure.

In our study, we found that although Dex had an elimination half-life of 2-3 h, patients received Dex treatment showed lower plasma Cor, E, NE and 5-HT level 24 h after the surgery. It might due to the protective effect of Dex during the surgery, which lead to relatively low level of plasma hormone after surgery. Moreover, it might reduce the stress and inflammatory response, and prevent the cardiac complications among adults undergoing surgery [9, 24]. Chi et al. found that intraoperatively Dex use could attenuates myocardial injury in off-pump coronary artery bypass graft surgery, and it could stabilize the HR even 48 h after surgery [30]. In our study, we found patients in Dex group had shorter ventilator time and hospitalization time, and it might also due to the protective effect of Dex during the surgery. As an α2 adrenergic receptor agonist, Dex has minimal effects on respiration compared with other drugs [31]. Moreover, Dex could attenuate the lung injury and inflammation due to causes such as ischemia-reperfusion and ventilator, which might benefit respiratory function and reduce ventilator time [32, 33]. With regard to hospitalization time, study reported that intraoperative use of Dex was associated with faster recovery in patients who underwent major spinal surgery [18]. In addition, less postoperative complications might also lead to shorter hospitalization time.

There are certain limitations of our study. First, the sample size in our study is comparatively small, which might negatively influence our data. Second, we observed that a significant increase of plasma E level at T3, which might be correlated to the continued postoperative pumping of E and stimulus of intubation, misleading the real physical values of plasma E. Third, samples could only be collected from the blood plasma, which reflects the real situation from a single perspective. Fourthly, we conducted our surgeries between 9 and 11 am. Although this period is after the peak of the plasma Cor level, the circadian rhythm of Cor could still be a factor affecting the results. Lastly, after Dex administration, there may be a significant decrease in the heart rate, which may lead to the failure of the double blinded design for this clinical trial.

## Conclusions

Patients underwent CPB developed elevation of Cor, E, NE and 5-HT during the surgery. Dexmedetomidine can reduce Cor, E, NE, and 5-HT elevation in plasma after CPB in patients undergoing cardiac valve replacement, which then alleviated the stress response of the body. Moreover, it could shorten the hospitalization time and ventilator support time in the ICU, and had a positive effect on patient rehabilitation.

## Supplementary information

**Additional file 1. **Comparison of plasma Cor, E, NE and 5-HT levels in the two groups (±SD, *n*=30)

## Data Availability

All data generated or analysed during this study are included in this published article and supplementary material 1.docx.

## Abbreviations

5-HT: Serotonin
ACT: Activated prothrombin time
ACTH: Adrenocorticotropic hormone
ASA: American Society of Anesthesiologists
BIS: Bispectral index
Cor: Cortisol
CPB: Cardiopulmonary bypass
CRH: Corticotropin releasing hormone
DA: Dopamine
Dex: Dexmedetomidine hydrochloride
E: Epinephrine
ECMO: Extracorporeal membrane oxygenation
ELISA: Enzyme-linked immunosorbent assay
GC: Glucocorticoid
Hct: Hematocrit
HPA: Hypothalamic-pituitary-adrenal cortex
HPLC: High Performance Liquid Chromatography
HR: Heart rate
ICU: Intensive care unit
MAP: Mean arterial pressure
NE: Norepinephrine
α2-AR: α2 adrenergic receptor agonist
