# Supplementary material for: Effects of dexmedetomidine on stress hormones in patients undergoing cardiac valve replacement: a randomized controlled trial
Source: BMC Anesthesiol. 2020 Jun 6;20:142. doi: 10.1186/s12871-020-00993-0 (PMC7275125; doi:10.1186/s12871-020-00993-0)
Supplement: Supplementary file 1 — Additional file 1. Comparison of plasma Cor, E, NE and 5-HT levels in the two groups (±SD, n=30) [file 12871_2020_993_MOESM1_ESM.docx]

Supplementary material 1. Comparison of plasma Cor, E, NE and 5-HT levels in the two groups (**$\bar{X}$**±SD, n=30)

| Index | Group | T0 | T1 | T2 | T3 |
| --- | --- | --- | --- | --- | --- |
| Cor (ng/mL) | Group C | 24.18±9.95 | 38.58±6.90^a^ | 42.47±6.41^ab^ | 44.21±7.45^ab^ |
|  | Group D | 25.57±9.34 | 31.56±5.84^a*^ | 34.55±7.50^a*^ | 36.66±14.14^a*^ |
| E (pg/μL) | Group C | 2.01±1.68 | 2.68±1.95 | 3.04±2.39 | 3.17±2.22^a^ |
|  | Group D | 1.75±1.40 | 1.57±1.24^*^ | 1.71±1.22^*^ | 2.10±1.66^*^ |
| NE (pg/μL) | Group C | 2.75±2.02 | 3.66±1.94^a^ | 5.52±2.22^ab^ | 12.11±6.31^abc^ |
|  | Group D | 2.22±1.22 | 2.66±1.39^a*^ | 3.93±3.12^ab*^ | 8.35±5.55^abc*^ |
| 5-HT (pg/μL) | Group C | 4.85±3.12 | 3.74±2.98 | 8.19±2.81^ab^ | 10.73±5.5^abc^ |
|  | Group D | 4.61±4.08 | 3.02±1.63 | 6.77±2.63^ab*^ | 8.01±4.72^ab*^ |

T0: baseline; T1: at sternum sawing; T2: at end of extracorporeal circulation; T3: 24 hours after operation.

* *P* < 0.05 as compared with group C.

*^a^ P* < 0.05 as compared with T0.

*^b^ P* < 0.05 as compared with T1.

*^c^ P* < 0.05 as compared with T2.
